# Cardiovascular Mortality Can Be Predicted by Heart Rate Turbulence in Hemodialysis Patients

**DOI:** 10.3389/fphys.2020.00077

**Published:** 2020-02-11

**Authors:** Matthias C. Braunisch, Christopher C. Mayer, Axel Bauer, Georg Lorenz, Bernhard Haller, Konstantinos D. Rizas, Stefan Hagmair, Lukas von Stülpnagel, Wolfgang Hamm, Roman Günthner, Susanne Angermann, Julia Matschkal, Stephan Kemmner, Anna-Lena Hasenau, Isabel Zöllinger, Dominik Steubl, Johannes F. Mann, Thomas Lehnert, Julia Scherf, Jürgen R. Braun, Philipp Moog, Claudius Küchle, Lutz Renders, Marek Malik, Georg Schmidt, Siegfried Wassertheurer, Uwe Heemann, Christoph Schmaderer

**Affiliations:** ^1^Abteilung für Nephrologie, Klinikum Rechts der Isar, Fakultät für Medizin, Technische Universität München, Munich, Germany; ^2^Center for Health & Bioresources, Biomedical Systems, AIT Austrian Institute of Technology GmbH, Vienna, Austria; ^3^Department of Cardiology, Munich University Clinic, German Centre for Cardiovascular Research, Ludwig Maximilian University of Munich, Munich, Germany; ^4^University Hospital for Internal Medicine III, Medical University Innsbruck, Innsbruck, Austria; ^5^Institut für Medizinische Informatik, Statistik und Epidemiologie, Klinikum Rechts der Isar, Fakultät für Medizin, Technische Universität München, Munich, Germany; ^6^Department of Nephrology, University of Erlangen-Nuremberg, Erlangen, Germany; ^7^KfH Kidney Center, Munich, Germany; ^8^Dialysis Center Munich Nord, Munich, Germany; ^9^Praxen Dr. Braun, Dialysis Center, Dingolfing, Germany; ^10^National Heart and Lung Institute, Imperial College London, London, United Kingdom; ^11^Klinik für Innere Medizin I, Klinikum Rechts der Isar, Fakultät für Medizin, Technische Universität München, Munich, Germany

**Keywords:** heart rate turbulence, cardiovascular mortality, baroreflex, mortality risk prediction, hemodialysis

## Abstract

**Background:**

Excess mortality in hemodialysis patients is mostly of cardiovascular origin. We examined the association of heart rate turbulence (HRT), a marker of baroreflex sensitivity, with cardiovascular mortality in hemodialysis patients.

**Methods:**

A population of 290 prevalent hemodialysis patients was followed up for a median of 3 years. HRT categories 0 (both turbulence onset [TO] and slope [TS] normal), 1 (TO or TS abnormal), and 2 (both TO and TS abnormal) were obtained from 24 h Holter recordings. The primary end-point was cardiovascular mortality. Associations of HRT categories with the endpoints were analyzed by multivariable Cox regression models including HRT, age, albumin, and the improved Charlson Comorbidity Index for hemodialysis patients. Multivariable linear regression analysis identified factors associated with TO and TS.

**Results:**

During the follow-up period, 20 patients died from cardiovascular causes. In patients with HRT categories 0, 1 and 2, cardiovascular mortality was 1, 10, and 22%, respectively. HRT category 2 showed the strongest independent association with cardiovascular mortality with a hazard ratio of 19.3 (95% confidence interval: 3.69–92.03; *P* < 0.001). Age, calcium phosphate product, and smoking status were associated with TO and TS. Diabetes mellitus and diastolic blood pressure were only associated with TS.

**Conclusion:**

Independent of known risk factors, HRT assessment allows identification of hemodialysis patients with low, intermediate, and high risk of cardiovascular mortality. Future prospective studies are needed to translate risk prediction into risk reduction in hemodialysis patients.

## Introduction

End-stage renal disease patients treated with chronic dialysis are at high mortality risk, frequently succumbing to cardiovascular death (Foley et al., 1998). The incidence of sudden cardiac death is particularly high compared to patients with ischemic heart disease (USRDS, 2014). These patients are often of advanced age and suffer from multiple comorbidities (Ortiz et al., 2011). Standard risk factors including high cholesterol do not predict mortality (Fellström et al., 2009; Chan K.E. et al., 2010). Uremia affects autonomous nervous system in end-stage renal disease, and sympathetic overactivity has consistently been reported (Stenvinkel et al., 2008; Chan C.T. et al., 2010; Rubinger et al., 2013). Consequently, standard autonomic tests, e.g., conventional time- and frequency-domain parameters of heart rate variability (HRV), are blunted in renal patients compared to healthy controls (Celik et al., 2011) but fail to predict mortality in adjusted models (Suzuki et al., 2012). At the same time, cardiovascular and sudden cardiac death risk stratification of end-stage renal patients is presently an important unmet clinical need. Blanket prophylaxis by implantable defibrillators does not improve survival in these patients (Jukema et al., 2019).

Risk stratification of ischemic heart disease patients has been substantially improved by novel autonomic tests including heart rate turbulence (HRT) (Barthel et al., 2003; Bauer et al., 2009). In post-myocardial infarction patients, HRT category 2 was the strongest independent predictor of mortality amongst other risk factors including age ≥ 65 years, diabetes mellitus and left ventricular ejection fraction (LVEF) ≤ 30% (Barthel et al., 2003). Furthermore, a combination of HRT category 2 and abnormal cardiac deceleration capacity identified high-risk post-myocardial infarction patients with LVEF > 30%. The risk in these patients was nearly as high as the risk in those with LVEF ≤ 30% (Bauer et al., 2009).

HRT refers to sinus cycle length fluctuations in response to spontaneous premature ventricular contractions (PVC) (Bauer et al., 2008). Under the condition of normal autonomic function, there is a transient rate acceleration phase of vagal withdrawal immediately after the PVC compensatory pause. This is subsequently followed by a gradual rate deceleration phase due to parasympathetic restoration after sympathetic-mediated gradual increase of arterial resistance. Abnormal autonomic status does not show these HRT variations (Bauer et al., 2009). The components of HRT, namely turbulence onset (TO) and turbulence slope (TS), correlate with baroreflex sensitivity (Wichterle et al., 2002; Iwasaki et al., 2005) thus expressing essential mechanisms of hemodynamic stability maintenance.

Hemodynamic stability during dialysis is important to avoid intradialytic hypotension, which has been associated with impaired sympathovagal balance (Rubinger et al., 2004) and increased mortality (Flythe et al., 2015). In multivariable analysis, an independent association of HRT with all-cause mortality could not be proven in hemodialysis patients (Suzuki et al., 2012). So far, however, HRT-based prediction of cardiovascular mortality has not been reported in hemodialysis patients.

Consequently, the aims of the present study were to examine HRT in a large cohort of unselected, prevalent hemodialysis patients and to study its association with cardiovascular mortality. Specifically, the study reported here hypothesized that HRT is associated with cardiovascular mortality independent of known risk factors. Risk prediction using HRV and HRT parameters was a prospectively defined goal of the study (Schmaderer et al., 2016).

## Materials and Methods

### Study Population

The study investigated the “rISk strAtification in end-stage Renal disease”- (ISAR)-cohort, a multicenter, prospective longitudinal observational cohort study (ClinicalTrials.gov; identifier number: NCT01152892) (Schmaderer et al., 2016). The study protocol, conforming to the ethical guidelines of the Helsinki Declaration, was approved by the Medical Ethics Committee of the Klinikum rechts der Isar of the Technical University Munich and of the Bavarian State Board of Physicians. Patients were recruited from 17 hemodialysis centers in the greater Munich area between April 2010 and January 2014. All participants gave informed written consent. Inclusion criteria were age ≥ 18 years and dialysis vintage ≥ 90 days (Schmaderer et al., 2016). Patients were excluded if pregnant or if suffering from ongoing infection or malignancy with a life expectancy ≤ 24 months (Schmaderer et al., 2016). Out of the 519 patients meeting inclusion criteria, 390 consented to undergo 24 h Holter electrocardiogram (ECG) recording. Out of these patients, 100 had to be excluded (11 and 45 suffered from paroxysmal and permanent atrial fibrillation, respectively, 27 had a pacemaker implanted, and 17 showed < 75% of normal sinus rhythm beats).

### Clinical Characteristics

Demographic and clinical data were obtained at baseline. Baseline comorbidities were assessed using an adapted version of the Charlson Comorbidity Index that has been validated for mortality prediction in hemodialysis patients (Liu et al., 2010). The Index assigns numerical weights to the comorbid conditions of atherosclerotic heart disease (1), heart failure (3), cerebrovascular accident/transient ischemic attack (2), peripheral vascular disease (2), dysrhythmia (2), other cardiac disease (2), chronic obstructive pulmonary disease (2), gastrointestinal bleeding (2), liver disease (2), cancer (2), and diabetes (1). A comorbidity score of a patient is the sum of the assigned numerical weights and ranges between 0 and 21 (Liu et al., 2010). Blood samples were obtained prior to a midweek dialysis session at baseline. Serum chemistry analyses were performed by ISO (International Organization for Standardization) certified local laboratories of the dialysis centers.

### Endpoints

Mortality was assessed after a median of 3 years by medical reports, databases of each dialysis center, or by contacting the attending physician or the next of kin. Using this information, the ISAR Endpoint Committee classified the underlying cause of death in each case (Schmaderer et al., 2016). Cardiovascular mortality was considered as the primary endpoint and all-cause mortality as the secondary endpoint.

### HRT and HRV Calculation

The 24 h 12-lead ECGs (1000 Hz) were recorded using the Lifecard CF digital Holter recorder (Delmar Reynolds/Spacelabs Healthcare, Nuremberg, Germany) and started before a midweek dialysis session. Reference ECG annotations and RR-interval measurements were performed using the software tools of the equipment (Pathfinder, Delmar Reynolds/Spacelabs Healthcare, Nuremberg, Germany; v.9.027) (Molgaard, 1991). An experienced physician, blinded to the clinical status, visually reviewed and, where appropriate, manually corrected the computerized RR measurements. Calculation of HRT was performed as previously described, if ≥ 5 PVCs were present in the complete recording (Bauer et al., 2008). In brief, HRT category 0 was defined as TO (<0%) and TS (>2.5 ms/R-R interval) both normal or fewer than 5 PVCs present (Barthel et al., 2003; Bauer et al., 2009). HRT category 1 was defined as either TO or TS abnormal, and HRT category 2 was defined as both TO and TS abnormal. Traditional HRV parameters of the time-domain and frequency-domain were computed using the 24 h RR interval series according to established standards (Camm et al., 1996). Logarithmic transformation was additionally performed for frequency-domain parameters. Additional non-linear HRV parameters were calculated as previously described (Peng et al., 1995; Penzel et al., 2003; Bauer et al., 2006; Suzuki et al., 2012). Severe autonomic failure (SAF) was assumed if HRT category 2 and abnormal deceleration capacity (≤4.5 ms) were present (Bauer et al., 2009).

### Statistical Analysis

Categorical data are presented as absolute and relative frequencies. Continuous variables are expressed as mean ± standard deviation (SD) for normally distributed variables and as median and interquartile range (IQR) for variables with skewed distribution. Group differences are assessed by χ^2^ test for categorical variables and one-way analysis of variance (ANOVA) or Kruskal–Wallis test for continuous variables, as appropriate.

Cumulative incidence functions of the cardiovascular death probability were computed. Cause-specific hazard for cardiovascular mortality were compared between groups by the log-rank test. Median follow-up time was assessed using the reverse Kaplan–Meier method. Univariate Cox proportional hazards regression analysis of HRV parameters was performed for the endpoints. Multivariable Cox proportional hazards model included adjustment variables with previously reported strong links to mortality in hemodialysis patients (Stenvinkel et al., 2008; Herselman et al., 2010; Ortiz et al., 2011; Suzuki et al., 2012). Two multivariable models for the association with cardiovascular mortality were considered: Model 1 included age and albumin, Model 2 included age and the Charlson Comorbidity Index. For all-cause mortality further variables were added to these models. Model 3 added high-sensitivity C-reactive protein (hsCRP) and calcium phosphate product (CaP) to Model 1, comparable to Suzuki et al. (2012). In Model 4, the Charlson Comorbidity Index was added to Model 3. Further, exploratory models were also tested (Supplementary Tables 4, 5).

Multivariable linear regression analysis using backward selection was used to identify factors associated with TO and TS. All variables from Table 1, except the Charlson Comorbidity Index (to identify specific associated comorbid conditions) and heart rate, were considered as potential predictors. To retain constant numbers of patients within the Cox regression models, 12 missing hsCRP values were replaced by non-hsCRP values and 3 total calcium values were imputed as dialysis center-specific means.

**TABLE 1:** Overall baseline characteristics and grouped by categories 0 to 2 of heart rate turbulence (HRT).

Parameter	Total group (*n* = 290)	Heart rate turbulence (HRT) category			*P*
		Category 0 (*n* = 161)	Category 1 (*n* = 88)	Category 2 (*n* = 41)	
Age [years]	63.9 [50.7–73.8]	56.1 [45.4–67.9]	72.0 [62.2–78.3]	70.8 [62.9–75.7]	< 0.001
Gender [male]	190 (65.5)	107 (66.5)	59 (67.0)	24 (58.5)	0.60
Body mass index [kg/m^2^]	25.0 [22.4–28.7]	24.5 [22.4–28.2]	26.0 [22.8–29.1]	25.6 [22.4–29.3]	0.096
Dialysis vintage [months]	46.0 [24.0–78.3]	50.0 [21.5–85.5]	45.5 [29.0–75.8]	40.0 [25.0–74.5]	0.73
Ultrafiltration rate [ml/h]	483.0 ± 259.5	495.2 ± 266.9	466.6 ± 246.7	469.8 ± 260.6	0.67
Net ultrafiltration [l]	1.7 ± 1.2	1.8 ± 1.2	1.5 ± 1.1	1.5 ± 1.1	0.16
Kt/V	1.46 [1.21–1.65]	1.45 [1.21–1.74]	1.45 [1.20–1.62]	1.48 [1.31–1.64]	0.68
Systolic blood pressure [mmHg]	137.0 [123.0–149.0]	136.0 [123.0–149.0]	138.5 [122.8–149.3]	137.0 [124.5–151.8]	0.71
Diastolic blood pressure [mmHg]	75.1 ± 14.6	76.2 ± 14.5	73.1 ± 14.3	75.1 ± 15.3	0.29
Heart rate [bpm]	73.7 [65.6–80.9]	74.6 [66.6–81.8]	72.8 [64.6–79.7]	73.4 [65.1–81.1]	0.50
Blood urea nitrogen [mg/dl]	62.8 ± 16.5	64.9 ± 17.0	60.1 ± 16.3	60.5 ± 13.5	0.055
Phosphate [mmol/l]	1.74 ± 0.49	1.73 ± 0.46	1.70 ± 0.49	1.83 ± 0.56	0.40
Total calcium [mmol/l]	2.27 ± 0.18	2.26 ± 0.18	2.28 ± 0.18	2.28 ± 0.18	0.64
Calcium *x* phosphate [mmol^2^/l^2^]	3.93 ± 1.12	3.90 ± 1.09	3.89 ± 1.14	4.12 ± 1.23	0.49
Creatinine [mg/dl]	8.81 ± 2.83	9.47 ± 2.93	8.02 ± 2.51	7.95 ± 2.41	< 0.001
High-sensitivity CRP [mg/dl]	0.38 [0.16–0.90]	0.31 [0.16–0.77]	0.48 [0.19–1.00]	0.57 [0.16–1.01]	0.023
Albumin [g/dl]	4.02 ± 0.40	4.06 ± 0.41	3.96 ± 0.42	3.96 ± 0.30	0.12
Parathyroid hormone [pg/ml]	238.5 [127.4–402.9]	246.0 [109.0–402.3]	241.5 [128.2–404.3]	232.6 [144.1–387.3]	0.98
Leukocytes [G/l]	6.97 ± 2.06	6.95 ± 2.07	6.64 ± 1.84	7.75 ± 2.34	0.018
Total cholesterol [mg/dl]	177.0 [154.0–207.0]	179.0 [152.8–206.3]	172.0 [148.0–202.0]	181.0 [159.5–222.5]	0.45
Charlson Comorbidity Index [0–21]	2.0 [1.0–5.0]	2.0 [0.0–4.0]	4.0 [2.0–5.0]	4.0 [2.0–6.0]	< 0.001
Diabetes mellitus	99 (34.1)	49 (30.4)	30 (34.1)	20 (48.8)	0.087
History of myocardial infarction	47 (16.2)	17 (10.6)	23 (26.1)	7 (17.1)	0.006
Left ventricular hypertrophy	76 (26.2)	41 (25.5)	23 (26.1)	12 (29.3)	0.89
Heart failure	31 (10.7)	10 (6.2)	16 (18.2)	5 (12.2)	0.013
Peripheral artery disease	55 (19.0)	20 (12.4)	19 (21.6)	16 (39.0)	< 0.001
Hypertension	271 (93.4)	145 (90.1)	86 (97.7)	40 (97.6)	0.034
Coronary heart disease	88 (30.3)	37 (23.0)	35 (39.8)	16 (39.0)	0.010
Cerebral vascular disease	41 (14.1)	12 (7.5)	16 (18.2)	13 (31.7)	< 0.001
Smoking [ever]	79 (27.4)	47 (29.6)	21 (23.9)	11 (26.8)	0.63
Antihypertensive medication	258 (89.0)	137 (85.1)	81 (92.0)	40 (97.6)	0.041
Statin	104 (36.4)	48 (30.2)	38 (44.2)	18 (43.9)	0.052

*n (%) for categorical data, mean ± standard deviation or median [interquartile range] for continuous data, as appropriate. CRP, c-reactive protein.*

All statistical tests were two-sided and *P*-values < 0.05 were considered significant. Statistical analysis was performed using SPSS version 25.0 (SPSS, Inc., Chicago, IL, United States) for Mac and R version 3.4.2. (R Foundation for Statistical Computing, Vienna, Austria).

## Results

### Patient Characteristics

**FIGURE 1:**
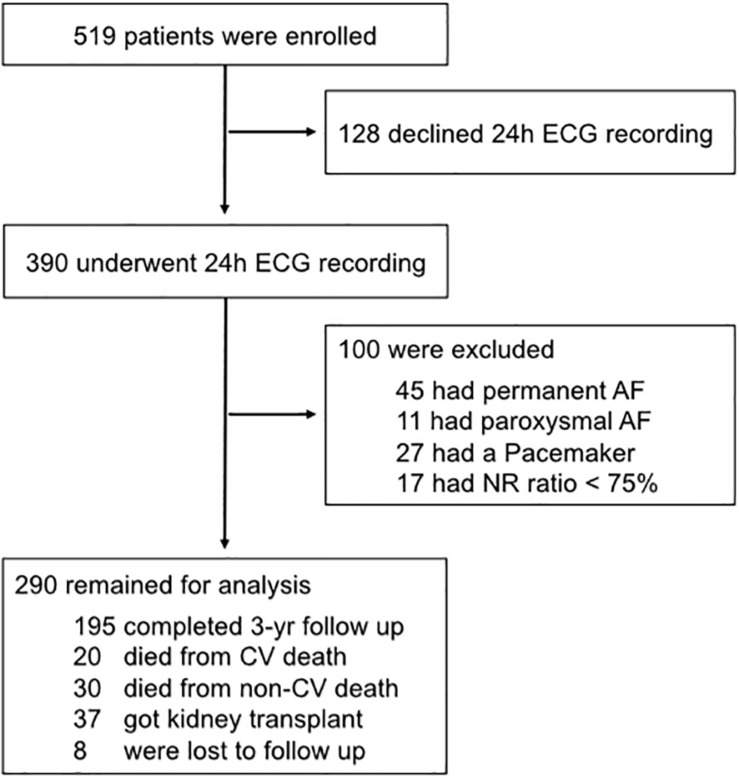
Flowchart of participants. Out of the 519 patients, 390 consented to undergo 24 h Holter electrocardiogram (ECG). Of these, 100 had to be excluded because of atrial fibrillation, a permanent pacemaker implant, or because of having less than 75% of normal sinus rhythm beats. 290 patients remained for the present analysis. AF, arterial fibrillation; CV, cardiovascular; ECG, electrocardiogram; NR, normal rhythm; yr, year.

In total, 290 patients with a 3-year median follow-up were eligible for the analysis (Figure 1). The median age was 64 [IQR 51–74] years, 34.5% of the patients were female. Of the 290 patients, 161 patients (55.5%) fell into HRT category 0 (including 108 patients with absent or fewer than 5 PVCs), 88 patients (30.3%) into HRT category 1, and 41 patients (14.2%) into HRT category 2. HRT category 0 and HRT category non-calculable were merged because of no significant difference in the survival probabilities between these patients (χ^2^ = 0.25, log-rank *P* = 0.62) (Supplementary Figure 1). Age, creatinine, Charlson Comorbidity Index, the intake of antihypertensive medication and comorbidities differed significantly between HRT categories 0 to 2 (Table 1 and Supplementary Table 1). Compared to the excluded patients of the ISAR study cohort, patients in the analyzed population were younger and had relatively fewer comorbidities (see Supplementary Table 2).

### Association of HRT and Mortality

**FIGURE 2:**
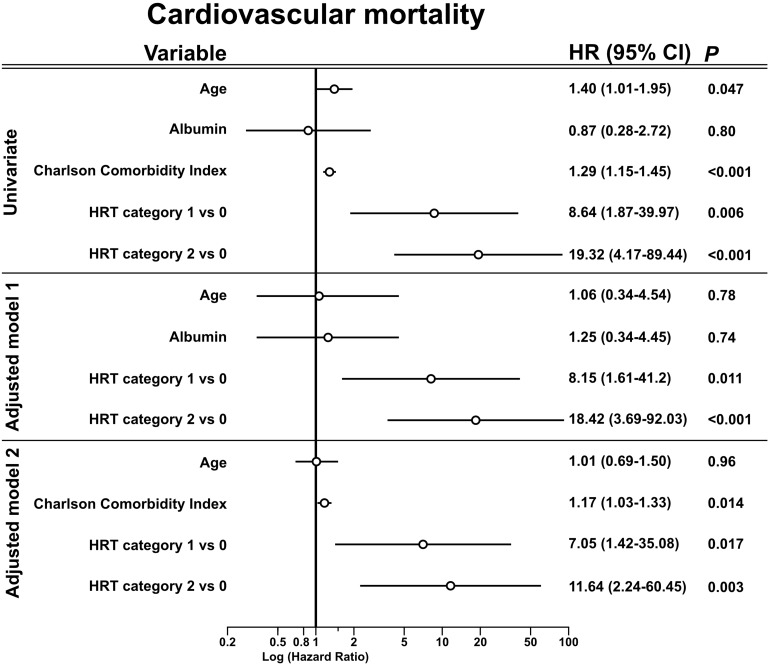
Association of risk variables with cardiovascular mortality in univariate and multivariable analysis. Univariate and multivariable Cox regression analysis for the association of the risk variable and cardiovascular mortality. Unadjusted and adjusted hazard ratios with the respective confidence interval are presented. Age has the unit 10 years. The adjusted Model 1 included age, albumin and HRT. The adjusted Model 2 included age, the Charlson Comorbidity Index and HRT. Units: Age, 10 years; Albumin, 1 g/dl; Charlson Comorbidity Index, 1 point. CI, confidence interval; HR, hazard ratio; HRT, heart rate turbulence.

**FIGURE 3:**
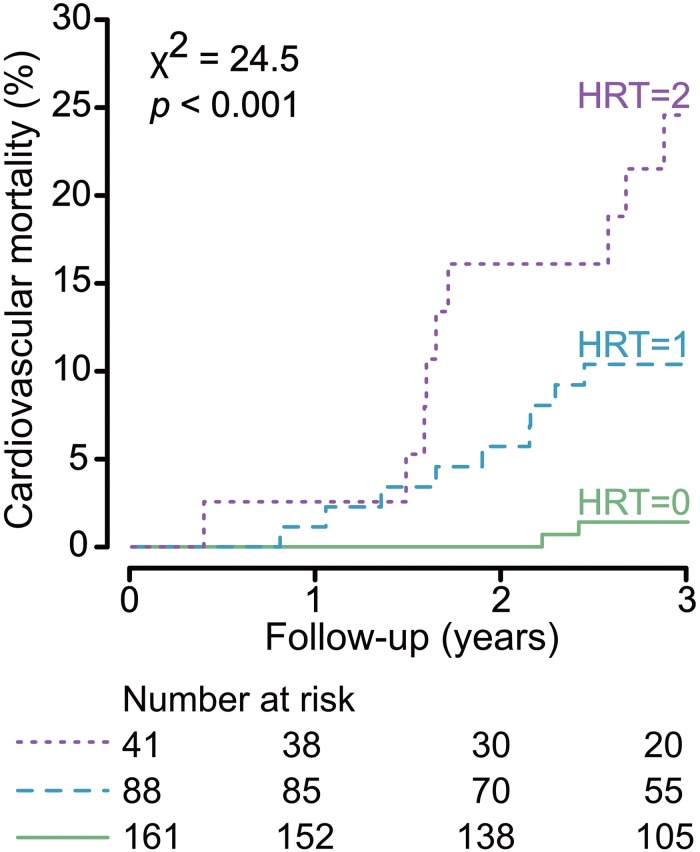
Cumulative cardiovascular mortality curves stratified by heart rate turbulence (HRT). The median follow-up was 3 years. Cause-specific hazards for cardiovascular mortality were compared between groups by the log-rank test. The number of patients of the individual groups involved in the analysis at 1, 2, and 3 years are shown under the graph. 3-Year cardiovascular mortality rates were 1.2, 10.2, and 22.0% for those with HRT category 0, 1, and 2, respectively.

Twenty patients suffered cardiovascular mortality: sudden cardiac death (*n* = 7), myocardial infarction (*n* = 2), heart failure (*n* = 5), major stroke (*n* = 2), cardiac surgical procedure (*n* = 1), aortal dissection (*n* = 1), ruptured aortic aneurysm (*n* = 1), and mesenteric ischemia (*n* = 1). Non-cardiovascular death (*n* = 30) causes included: infectious events (*n* = 16), cancer (*n* = 5), gastrointestinal bleeding (*n* = 1), diabetic coma (*n* = 1), and withdrawal from treatment (*n* = 1). No agreement on cause of death was reached and the cause of death was defined “unknown” in six cases. Patients were censored at the last day of dialysis in case of renal transplantation (*n* = 37) or if lost to follow-up (*n* = 8). Of the seven patients who succumbed to sudden cardiac death, *n* = 1, 3, and 3 showed HRT category 0, 1, and 2, respectively. Figure 2 shows unadjusted and adjusted hazard ratios of HRT for cardiovascular mortality. In univariate analysis, HRT categories 1 and 2 compared to category 0 were associated with a substantially higher cardiovascular mortality risk. Supplementary Table 3 shows univariate hazard ratios of baseline parameters. After adjustment in different models, HRT category 1 and 2 remained significantly associated with cardiovascular mortality. The hazard ratio of HRT category 2, with category 0 as reference, ranged from 11.64 to 18.42 depending on the model. Three-year cardiovascular mortality rates were 1.2, 10.2, and 22.0% for those with HRT category 0, 1, and 2, respectively (Figure 3). Additional models of adjustment did not materially change the results (Supplementary Tables 4, 5).

None of the traditional HRV parameters of the time-domain and frequency-domain as well as non-linear HRV parameters were related to cardiovascular mortality risk in a univariate or an adjusted model (Table 2 and Supplementary Table 6), except for deceleration capacity that showed an association with cardiovascular mortality in a univariate analysis. Results for SAF are shown in Supplementary Table 7. Table 3 and Supplementary Table 8 show the median and interquartile ranges of HRV measures.

**TABLE 2:** Unadjusted and adjusted hazards ratios of traditional heart rate variability measures (time- and frequency-domain) for cardiovascular mortality.

Cardiovascular mortality risk							
Parameter	Unit	Univariate		Adjusted Model 1		Adjusted Model 2	
		HR (95% CI)	*P*	HR (95% CI)	*P*	HR (95% CI)	*P*
SDNN	ms	0.99(0.98−1.01)	0.27	0.99(0.98−1.01)	0.38	1.00(0.98−1.02)	0.84
SDANN	ms	0.99(0.98−1.01)	0.38	0.99(0.98−1.01)	0.5	1.00(0.98−1.02)	0.92
RMSSD	ms	0.99(0.93−1.05)	0.66	0.98(0.92−1.04)	0.44	0.99(0.94−1.05)	0.81
pNN50	%	0.97(0.85−1.10)	0.6	0.95(0.83−1.08)	0.41	0.96(0.84−1.10)	0.54
HRVTI		0.96(0.91−1.02)	0.21	0.97(0.91−1.03)	0.36	0.99(0.94−1.05)	0.81
TINN	ms	1.00(1.00−1.00)	0.38	1.00(1.00−1.00)	0.61	1.00(1.00−1.00)	0.93
Total Power	ms^2^	1.00(1.00−1.00)	0.18	1.00(1.00−1.00)	0.24	1.00(1.00−1.00)	0.56
ULF	ms^2^	1.00(1.00−1.00)	0.22	1.00(1.00−1.00)	0.32	1.00(1.00−1.00)	0.65
VLF	ms^2^	1.00(1.00−1.00)	0.11	1.00(1.00−1.00)	0.17	1.00(1.00−1.00)	0.43
LF	ms^2^	1.00(1.00−1.00)	0.084	1.00(1.00−1.00)	0.094	1.00(1.00−1.00)	0.21
HF	ms^2^	1.00(1.00−1.00)	0.47	1.00(1.00−1.00)	0.26	1.00(1.00−1.00)	0.44
LF/HF		0.71(0.49−1.03)	0.073	0.79(0.53−1.16)	0.23	0.82(0.58−1.15)	0.82
DFA a_1_		0.37(0.08−1.71)	0.2	0.66(0.12−3.50)	0.62	0.74(0.15−3.66)	0.71
DFA a_2_		7.56(0.01−5465.10)	0.55	1.68(0.00−1618.04)	0.88	0.85(0.00−775.81)	0.96
DC category 1 vs. 0		0.67(0.27−1.69)	0.4	0.70(0.28−1.75)	0.44	0.87(0.34−2.24)	0.77
DC category 2 vs. 0		0.20(0.04−0.92)	< 0.05	0.26(0.05−1.24)	0.09	0.44(0.09−2.20)	0.32
AC	ms	1.19(0.93−1.52)	0.16	1.17(0.91−1.50)	0.22	1.06(0.84−1.34)	0.63

*Model 1: age, albumin; model 2: age, Charlson Comorbidity Index. CI, confidence interval; SDNN, standard deviation of all NN intervals; SDANN, standard deviation of the averages of NN intervals in all 5-min segments of 24 h recording; RMSSD, square root of the mean square of differences between adjacent NN intervals; pNN50, adjacent NN intervals differing ¿ 50 ms in the total NN intervals; HRVTI, HRV triangular index: number of NN intervals over the number of NN-intervals in the modal bin; TINN, triangular interpolation of NN interval histogram; ULF, ultra-low frequency; VLF, very low frequency; LF, low frequency; HF, high frequency; LF/HF, LF to HF ratio; DFA, detrended fluctuation analysis; DC, deceleration capacity; AC, acceleration capacity.*

**TABLE 3:** Measures of heart rate variability at baseline in patients overall and grouped by survival state.

Parameter	Unit	Overall	Cardiovascular mortality	
			Survivor (*n* = 270)	Non-survivor (*n* = 20)
SDNN	ms	86.8(69.0−105.6)	87.0(69.1−105.9)	85.6(64.7−99.5)
SDANN	ms	77.5(61.5−97.6)	77.6(62.3−98.2)	78.7(56.1−94.1)
RMSSD	ms	13.6(10.0−20.5)	13.6(10.2−20.6)	13.9(9.2−19.7)
pNN50	%	0.86(0.18−3.07)	0.86(0.18−3.14)	0.90(0.27−2.35)
HRVTI		22.5(17.9−27.9)	22.6(18.0−28.1)	21.3(16.8−26.5)
TINN	ms	346.4(272.0−432.1)	347.5(272.0−433.0)	327.7(264.4−414.3)
Total Power	ms^2^	7803.3(4912.0−11864.1)	8018.5(4994.0−12065.6)	6893.6(4396.9−10796.9)
ULF	ms^2^	6466.3(3829.6−10138.6)	6513(4014.0−10171.6)	5426.0(3444.7−9695.3)
VLF	ms^2^	677.0(383.8−1123.6)	682.6(393.3−1154.1)	569.6(297.3−843.4)
LF	ms^2^	270.4(137.2−510.7)	285.8(143.7−528.2)	160.6(130.0−274.3)
HF	ms^2^	109.7(51.3−254.6)	116.9(51.1−225.9)	89.3(59.6−179.6)
LF/HF		2.1(1.5−3.5)	2.1(1.5−3.5)	1.7(1.3−2.1)
DFA a_1_		1.25(1.06−1.48)	1.25(1.07−1.49)	1.18(0.98−1.39)
DFA a_2_		1.14(1.10−1.19)	1.14(1.10−1.18)	1.15(1.10−1.21)
DC	ms	3.36(2.37−4.86)	3.48(2.44−5.03)	2.93(1.76−4.10)
AC	ms	−4.17(−5.92*to*−3.03)	−4.24(−5.95*to*−3.05)	−3.54(−4.94*to*−2.40)

*Values are expressed as median (interquartile range). SDNN, standard deviation of all NN intervals; SDANN, standard deviation of the averages of NN intervals in all 5-min segments of 24 h recording; RMSSD, square root of the mean square of differences between adjacent NN intervals; pNN50, adjacent NN intervals differing ¿ 50 ms in the total NN intervals; HRVTI, HRV triangular index: number of NN intervals over the number of NN-intervals in the modal bin; TINN, triangular interpolation of NN interval histogram; ULF, ultra-low frequency; VLF, very low frequency; LF, low frequency; HF, high frequency; LF/HF, LF to HF ratio; DFA detrended fluctuation analysis; DC, deceleration capacity; AC, acceleration capacity.*

Table 4 shows the unadjusted and adjusted hazard ratios of HRT for all-cause mortality. In univariate analysis, HRT categories were associated with all-cause mortality. All-cause mortality rates during the first 3 years were 8.7, 26.1, and 31.7% for those with HRT category 0, 1, and 2, respectively (*P* < 0.001, χ^2^ = 17.6). After adjustment according to Model 3, HRT category 2, with category 0 as reference, remained significantly and independently associated with all-cause mortality (Table 4). However, after adding the Charlson Comorbidity Index to the model (adjusted Model 4), categorical HRT was no longer significantly associated with all-cause mortality risk.

**TABLE 4:** Association of risk variables with all-cause mortality in univariate and multivariable analysis.

		Univariate		Adjusted Model 3		Adjusted Model 4	
Variable	Unit	HR (95% CI)	*P*	HR (95% CI)	*P*	HR (95% CI)	*P*
Age	10 year	1.56(1.25−1.95)	0.001	1.31(1.02−1.67)	0.033	1.25(0.97−1.60)	0.86
Albumin	1 g/dl	0.23(0.11−0.45)	0.001	0.29(0.13−0.62)	0.002	0.33(0.15−0.74)	0.007
High-sensitivity CRP	1 mg/dl	1.26(1.13−1.39)	0.001	1.20(1.07−1.35)	0.002	1.18(1.04−1.33)	0.008
Calcium *x* phosphate	mmol^2^/l^2^	0.99(0.77−1.27)	0.91	1.01(0.77−1.33)	0.95	0.98(0.74−1.31)	0.91
Charlson Comorbidity Index	1 point	2.14(1.65−2.78)	0.001	−	−	1.20(1.10−1.31)	0.001
HRT category 1 vs. 0		3.13(1.61−6.09)	0.001	1.98(0.97−4.03)	0.061	1.76(0.87−3.56)	0.12
HRT category 2 vs. 0		3.93(1.85−8.36)	0.001	2.86(1.31−6.27)	0.009	1.84(0.80−4.22)	0.15

*CI, confidence interval; CRP, c-reactive protein; HR, hazard ratio; HRT, heart rate turbulence.*

### Analysis of Determinants of TO and TS as Factors of HRT

In HRT category 1, 22 (25.0%) patients had an abnormal TO and 66 (75.0%) patients had an abnormal TS. For TO, age, CaP, and smoking status remained in the final model. Age, CaP, smoking status, and diastolic blood pressure were identified as the most relevant determinants for TS (Table 5).

**TABLE 5:** Multivariable linear regression with backward selection to identify determinants of TO and TS.

	Turbulence onset (TO) per 1%		Turbulence slope (TS) per ms	
Parameter	*b* (95% CI)	*P*	*b* (95% CI)	*P*
Intercept	−5.14(−7.31*to*−2.97)	< 0.001	22.23(14.49*to*−30.00)	< 0.001
Age [10 years]	0.46(0.21−0.70)	< 0.001	−0.19(−2.45*to*−1.28)	< 0.001
Calcium *x* phosphate [1 mmol^2^/l^2^]	0.33(0.01−0.65)	0.042	−0.74(−1.45*to*−0.03)	0.042
Smoking	0.93(0.11−1.74)	0.026	−2.15(−3.95*to*−0.34)	0.020
Diabetes mellitus	−	−	−2.28(−3.90*to*−0.67)	0.006
Diastolic blood pressure [1 mmHg]	−	−	−0.06(−0.11*to*−0.00)	0.039
Blood urea nitrogen [1 mg/dl]	−	−	0.04(−0.01*to*0.09)	0.096

*Dependent variables in each model: TO and TS. *R*^2^ (TO model) = 0.09; *R*^2^ (TS model) = 0.24. *b*, regression coefficient; CI, confidence interval.*

## Discussion

The results of this observational analysis demonstrate that HRT is independently and strongly associated with cardiovascular mortality in prevalent hemodialysis patients. Approximately one half of the patients of our cohort had HRT category 1 and 2 and displayed an intermediate or high 3-year cardiovascular mortality risk of 10 and 22%, respectively. The other half of the patients with HRT category 0 had a much lower 3-year cardiovascular mortality risk of only 1%. Adding multiple risk variables for mortality in hemodialysis patients to the Cox regression, like age, albumin or multiple comorbidities, the hazard ratio of HRT categories decreased only slightly with HRT category 2 showing the highest hazard ratios. Of particular interest is the subgroup of patients with HRT category 0 who accounted for more than a half of the study population and who displayed a very low risk of cardiovascular mortality over 3 years. It appears that in these patients, examinations and interventions going beyond standard care of dialysis patients to prevent cardiovascular mortality might be unnecessary.

Additionally, we showed independent association between HRT and all-cause mortality. However, similar to previous studies in hemodialysis patients (Suzuki et al., 2012), after adding the Charlson Comorbidity Index for multiple comorbidities to the fully adjusted model, HRT was no longer independently associated with all-cause mortality.

Similar to previous reports (Suzuki et al., 2012), we have not found standard and non-linear HRV parameters helpful in predicting cardiovascular mortality. The observed association of SAF with cardiovascular mortality was attributable mainly to the high hazard ratio of HRT category 2 because deceleration capacity was not predictive in the multivariate models.

### Determinants of HRT in Hemodialysis Patients

Since impaired HRT is a consequence of baroreflex dysfunction, our results highlight the importance of complex sympathovagal interaction in the context of cardiovascular mortality risk prediction in hemodialysis patients. Multivariable regression identified an association of age, calcium phosphate product, and smoking status with TO and TS. Diabetes mellitus and diastolic blood pressure were only associated with TS. Both components of HRT, TO and TS, have been previously associated to baroreflex sensitivity (Davies et al., 2001). Autonomic control and parameters of HRT were reported worse in non-hemodialysis patients with older age, diabetes mellitus, hypertension or positive smoking status (Schroeder et al., 2003; Schwab et al., 2005; Bauer et al., 2008; Cagirci et al., 2009). The association of TS and diabetes mellitus is in line with previous publications suggesting that mainly TS is impaired by hyperglycemia mediated cardiac autonomic neuropathy which affects the parasympathetic restoration (i.e., TS) after a PVC (Balcioglu et al., 2007). In post-myocardial infarction patients with and without diabetes mellitus, HRT was associated in both groups with cardiac mortality (Miwa et al., 2011). However, especially in patients with LVEF > 30%, ≥ 65 years of age, the presence of diabetes mellitus and HRT category 2 showed a considerable elevated mortality risk compared to non-diabetic patients (Barthel et al., 2003).

Elevated CaP might be a dialysis-specific parameter with influence on HRT. It has been previously associated with an increased relative risk of death due to cardiac events in hemodialysis patients (Ganesh et al., 2001). Reduced perfusion of vascular macro- and myocardial microcirculation occurs in hemodialysis patients because of increased phosphate and CaP. The association of CaP and the components of HRT may represent an end-organ damage by the disruption of the normal conduction system architecture as well as by vascular obstruction. Furthermore, intradialytic hypotension is considered to be one of the key factors of cardiovascular damage and signifies increased mortality risk in hemodialysis patients (Shoji et al., 2004). However, the association of intradialytic hypotension with HRT is presently unknown.

Although not identified in our cohort, inflammation could be a further determinant of impaired HRT. In hemodialysis patients, chronic inflammation is present (Stenvinkel and Alvestrand, 2002). This could not only aggravate the identified determinants of HRT but also have a separate and additional impact on HRT. An interaction of the inflammatory and autonomous nervous system exists via the cholinergic anti-inflammatory pathway (Pavlov et al., 2003). Vagus nerve afferent sensory fibers are signaling the brain the presence of inflammation (Goehler et al., 2000) which is also impairing autonomous nervous system function via the centrally mediated activation of hypothalamus-pituitary-adrenal axis, increased sympathetic nervous system activity, and direct reduction of parasympathetic nervous system activity (Pavlov et al., 2003). Vagus nerve efferent cardiac fibers might cause HRT displayed baroreflex dysfunction in hemodialysis patients as evident in the HRT findings. However, further studies are needed to evaluate inflammation induced impaired autonomous nervous system in hemodialysis patients before experimental stimulation to enhance parasympathetic activity with an implantable device might become a therapeutic option (Lohmeier and Iliescu, 2011).

### Clinical Implications

The independent association of HRT with cardiovascular mortality addresses the need for risk stratification to deal with the excess cardiovascular mortality in hemodialysis patients. Classifying patients allows more precise clinical decisions independent of age, protein-energy wasting or comorbidities. Interestingly, more than half of the patient (55%, *n* = 161) with HRT category 0 showed a very low risk of 3-year cardiovascular mortality. This could allow a less stringent monitoring of cardiovascular comorbidities in the subgroup. Therefore, categorization by HRT also allows focused close monitoring of cardiovascular comorbidities in those patients at intermediate or high risk.

In our cohort, 35% (*n* = 7) of those who succumbed to cardiovascular causes suffered from sudden cardiac death which might be attributed to an arrhythmogenic event and is thus potentially preventable by the implantation of a cardioverter defibrillator (ICD) (Di Lullo et al., 2016). However, a recent randomized controlled trial including dialysis patients with a LVEF ≥ 35% showed no advantage of prophylactic ICD implantation (Jukema et al., 2019). One explanation for this result might be provided by recent studies showing that fatal arrhythmias are more frequently related to bradyarrhythmias than to tachyarrhythmias. Although ICDs also have a pacing capability, the brady-/tachyarrhythmia balance might still affect the ICD prophylaxis in hemodialysis patients (Roy-Chaudhury et al., 2018; Sacher et al., 2018; Genovesi et al., 2019). Another reason for the infectivity of ICD implantation in hemodialysis patients includes electrolyte imbalances which increases the risk of ineffective shock therapy. Of our seven patients who succumbed due to sudden cardiac death, only one had a normal HRT. Perhaps the assessment of LVEF ≤ 30% and HRT category 2 might help to select the candidates for ICD prophylaxis, reflecting the considerably increased mortality in this subgroup among post-myocardial infarction patients (Barthel et al., 2003). However, as already mentioned, the mode of sudden cardiac death might be different in hemodialysis patients compared to the general population with prevalent heart disease where ventricular arrhythmias are the main cause of sudden cardiac death. Noteworthy, ICD implantation could be more beneficial in secondary than in primary prevention (Genovesi et al., 2019).

Not surprisingly, we found HRT to be influenced by classical determinant of the autonomic nervous system function such as age, diabetes mellitus and smoking status and by factors causing structural cardiovascular damage, such as the CaP and hypertension. Therefore, it might also be important to modulate factors that increase sympathetic or decrease parasympathetic nervous system activity. In high-risk hemodialysis patients, central sympatholytic drugs like clonidine or moxonidine might therefore be beneficial (Rubinger et al., 2013). Whereas ß-blocker therapy had no effect on HRT in patients after myocardial infarction (Lin et al., 2002; Barthel et al., 2003), its anti-arrhythmic properties as well as a tight control of potassium and calcium during hemodialysis treatment may help reducing sudden cardiac death risk (Di Lullo et al., 2016). Heavy smokers among hemodialysis patients have an up to 41% increased relative risk for all-cause mortality compared to non-smokers (Li et al., 2018). Smoking cessation might be one modifiable factor to improve autonomic nervous system dysfunction. Furthermore, invasive and therefore limited options to reduce sympathetic overdrive by carotid baroreceptor stimulation during non-dialysis periods or sympathetic denervation of renal arteries have to be evaluated (Grassi et al., 2012; Rubinger et al., 2013). Further larger studies are needed to evaluate if smoking cessation, a precise treatment of diabetes mellitus, arterial hypertension or dialysis specific parameters (e.g., CaP) have an impact on HRT-documented baroreflex dysfunction.

In patients with structural heart disease with left ventricular dysfunction but relatively preserved LVEF, a combination of HRT category 2 and the presence of non-sustained ventricular tachycardia allowed a better prediction of all-cause mortality and fatal arrhythmic events compared to the single parameters (Kinoshita et al., 2019). Further research is warranted to evaluate if ECG based risk prediction might be of use in hemodialysis patients as well.

### Limitations

Limitations of the study also need to be considered. First, we only found a small number of 20 cardiovascular death cases. Multivariable adjustment was therefore possible only for a limited number of variables. To avoid the risk of overfitting (Babyak, 2004) and to account for different parameters, two multivariable models for the association with cardiovascular mortality were created. We used the Charlson Comorbidity Index to include multiple comorbidities in just one variable. For all-cause mortality, 50 cases existed, thus allowing to add further variables to the models. Second, we had no data on LVEF for model adjustment. Third, the high level of frailty in our cohort limited the number of available ECG recordings because of limited compliance with 24 h recordings, the presence of atrial fibrillation, or pacemaker implant, and low recording quality. Consenting to participate in a 24 h ECG recording and having an analyzable ECG recording may already represent potentially biased and beneficial pre-selection. Fourth, the calculation of HRT based on PVCs already represents certain cardiac impairment. In our cohort, PVC tachograms could not be calculated in 37% of the patients and summarized under HRT category 0 due to similar survival probabilities which is comparable to other studies (Barthel et al., 2003; Suzuki et al., 2012). Although the ECG is a routinely used, inexpensive and non-invasive tool, PVC annotation might be difficult and time consuming, especially in long-term ECG recordings. Therefore, automatic ECG annotation algorithms that allow a more precise identification of PVCs are needed. Finally, participants of the study were from the greater Munich area (i.e., mainly white Caucasians) and represent a younger subgroup with fewer comorbidities from the original ISAR cohort. Generalization of our results to other ethnic groups and populations is consequently potentially problematic.

## Conclusion

We were able to show that HRT is independently associated with cardiovascular mortality in hemodialysis patients. Patients with HRT category 0, 1, and 2 showed 1, 10, and 22% 3-year cardiovascular mortality rate, respectively. Patients in HRT category 0 who accounted for more than half of the study population had a very low risk of cardiovascular mortality over 3 years. On the other hand, HRT category 2 showed a particularly strong association with cardiovascular mortality. This allows classifying cardiovascular mortality risk of hemodialysis patients into low, intermediate, and high-risk strata. However, lager sample sizes are desired to validate these findings. As HRT displays impaired baroreflex activity, this permits a pathologic explanation for the increased cardiovascular mortality in hemodialysis patients. Identifying patients at intermediate and high risk may allow the development of prevention strategies as well as targeted therapies.

## Data Availability Statement

The datasets for this manuscript are not publicly available because written informed consent did not include wording on data sharing. Reasonable requests to access the datasets should be directed to CS, Christoph.Schmaderer@mri.tum.de.

## Ethics Statement

This study involving human participants was reviewed and approved by the Medical Ethics Committee of the Klinikum Rechts der Isar of the Technical University Munich and of the Bavarian State Board of Physicians. The patients/participants provided their written informed consent to participate in this study.

## Author Contributions

MB, CM, and CS contributed to the research idea and study design. MB, CM, AB, and CS drafted the article. MB, CM, GL, KR, SH, WH, LS, RG, SA, JM, SK, A-LH, IZ, DS, JFM, TL, JS, and JB contributed to the data acquisition. MB, CM, AB, GL, BH, and CS contributed to the data analysis and the data interpretation. MB, CM, and BH contributed to the statistical analysis. AB, PM, CK, LR, MM, GS, SW, UH, and CS contributed to the supervision.

## Conflict of Interest

JFM reports personal fees from AstraZeneca, Amgen, Braun, ACI, Fresenius, Gambro, Medice, Lanthio, Sanifit, Relypsa, ZS Pharma; grants and personal fees from Celgene, Abbvie, NovoNordisk, Roche, Sandoz; grants from European Union and McMaster University Canada outside the submitted work. The results presented in this manuscript have not been published previously in whole or part, except in abstract format. The remaining authors declare that the research was conducted in the absence of any commercial or financial relationships that could be construed as a potential conflict of interest.
