# Supplementary material for: Cardiovascular Mortality Can Be Predicted by Heart Rate Turbulence in Hemodialysis Patients
Source: Front Physiol. 2020 Feb 11;11:77. doi: 10.3389/fphys.2020.00077 (PMC7027389; doi:10.3389/fphys.2020.00077)
Supplement: Supplementary file 1 [file Table_1.docx]

**Supplementary Data**

**Title:**

**Cardiovascular mortality can be predicted by heart rate turbulence in hemodialysis patients**

**Authors**: Matthias C. Braunisch, and Christopher C. Mayer, Axel Bauer, Georg Lorenz, Bernhard Haller, Konstantinos D. Rizas, Stefan Hagmair, Lukas von Stülpnagel, Wolfgang Hamm, Roman Günthner, Susanne Angermann, Julia Matschkal, Stephan Kemmner, Anna-Lena Hasenau, Isabel Zöllinger, Dominik Steubl, Johannes F. Mann, Thomas Lehnert, Julia Scherf, Jürgen R. Braun, Philipp Moog, Claudius Küchle, Lutz Renders, Marek Malik, Georg Schmidt, Siegfried Wassertheurer, Uwe Heemann, and Christoph Schmaderer

# Supplemental Data Table of Contents

**Supplemental Table 1.** Detailed intake of antihypertensive medications overall and by categories of heart rate turbulence (HRT) – *page 2*

**Supplemental Table 2.** Cohort description in relation to excluded patients and study cohort – *page 3*

**Supplemental Table 3.** Unadjusted hazards ratios of baseline parameters for cardiovascular mortality – *page 4*

**Supplemental Table 4.** Explorative analysis of risk variables with cardiovascular mortality – *page 5*

**Supplemental Table 5.** Exploratory association of HRT with cardiovascular mortality

– *page 6*

**Supplemental Table 6.** Unadjusted and adjusted hazards ratios of ln transformed frequency-domain heart rate variability measures for cardiovascular mortality **–** *page 7*

**Supplemental Table 7.** Association of severe autonomic failure (SAF) with cardiovascular mortality in univariate and multivariable analysis – *page 8*

**Supplemental Table 8.** Ln transformed frequency-domain measures of heart rate variability at baseline in patients overall and grouped by survival state – *page 9*

**Supplemental Figure 1.** Cumulative cardiovascular mortality curves stratified by heart rate turbulence (HRT) including HRT category non-calculable. – *page 10*

| **Supplemental Table 1.** Detailed intake of antihypertensive medications overall and by categories of heart rate turbulence (HRT) | | | | | |
| --- | --- | --- | --- | --- | --- |
| **Parameter** | **Total group**  **(n = 290)** | **Heart rate turbulence (HRT)** | | |  |
|  |  | **Category 0**  **(n = 161)** | **Category 1**  **(n = 88)** | **Category 2**  **(n = 41)** | ***P*** |
| Antihypertensive medication | 258 (89.0) | 137 (85.1) | 81 (92.0) | 40 (97.6) | 0.041 |
| - ACEI | 108 (37.2) | 62 (38.5) | 34 (38.6) | 12 (29.3) | 0.52 |
| - ARB | 71 (24.5) | 34 (21.1) | 23 (26.1) | 14 (34.1) | 0.20 |
| - CCB | 123 (42.4) | 70 (43.5) | 31 (35.2) | 22 (53.7) | 0.13 |
| - Vasodilator | 44 (15.2) | 24 (14.9) | 11 (12.5) | 9 (22.0) | 0.38 |
| - *ß*-blocker | 183 (63.1) | 93 (57.8) | 61 (69.3) | 29 (70.7) | 0.11 |
| - Diuretics | 170 (58.6) | 82 (50.9) | 61 (69.3) | 27 (65.9) | 0.011 |
| - Others | 41 (14.1) | 21 (13.0) | 12 (13.6) | 8 (19.5) | 0.56 |
| n (%) for categorical data. Abbreviations: ACEI, angiotensin-converting-enzyme inhibitor; ARB, angiotensin receptor blocker; CCB, calcium channel blocker. Others included drugs other than ACEI, ARB, CCB, Vasodilators, *ß*-blocker or diuretics (e.g. α-blocker, renin inhibitor). | | | | | |

| **Supplemental Table 2.** Cohort description in relation to excluded patients and study cohort | | | |
| --- | --- | --- | --- |
| **Parameter** | **Study population**  **(n = 290)** | **Excluded patients**  **(n = 229)** | ***P*** |
| Age [years] | 63.9 [50.7 - 73.8] | 73.0 [58.9 - 79.9] | <0.001 |
| Gender [male] | 190 (65.5) | 168 (73.4) | 0.06 |
| Body mass index [kg/m^2^] | 25.0 [22.4 - 28.7] | 25.5 [22.8 - 28.9] | 0.42 |
| Dialysis vintage [months] | 46.0 [24.0 - 78.3] | 41.0 [19.0 - 79.5] | 0.31 |
| Ultrafiltration rate [ml/h] | 483.0 ± 259.5 | 511.2 ± 265.2 | 0.22 |
| Net ultrafiltration [l] | 1.7 ± 1.2 | 1.9 ± 1.3 | 0.027 |
| Kt/V | 1.46 [1.21 - 1.65] | 1.46 [1.23 - 1.67] | 0.75 |
| Systolic blood pressure [mmHg] | 137.0 [123.0 - 149.0] | 132.0 [115.0 - 145.0] | 0.020 |
| Diastolic blood pressure [mmHg] | 75.1 ± 14.6 | 68.5 ± 14.7 | <0.001 |
| Heart rate [bpm] | 73.7 [65.6 - 80.9] | 71.9 [64.8 - 81.6] | 0.35 |
| Blood urea nitrogen [mg/dl] | 62.8 ± 16.5 | 59.2 ± 16.8 | 0.014 |
| Phosphate [mmol/l] | 1.74 ± 0.49 | 1.74 ± 0.58 | 0.85 |
| Total calcium [mmol/l] | 2.27 ± 0.18 | 2.26 ± 0.21 | 0.61 |
| Calcium *x* phosphate [mmol^2^/l^2^] | 3.93 ± 1.12 | 3.94 ± 1.36 | 0.98 |
| Creatinine [mg/dl] | 8.81 ± 2.83 | 7.98 ± 2.62 | 0.001 |
| High-sensitivity CRP [mg/dl] | 0.38 [0.16 - 0.90] | 0.62 [0.23 - 1.22] | 0.001 |
| Albumin [g/dl] | 4.02 ± 0.40 | 3.92 ± 0.42 | 0.007 |
| Parathyroid hormone [pg/ml] | 238.5 [127.4 - 402.9] | 184.9 [99.1 - 365.2] | 0.047 |
| Leukocytes [G/l] | 6.97 ± 2.06 | 6.94 ± 6.60 | 0.88 |
| Total cholesterol [mg/dl] | 177.0 [154.0 - 207.0] | 171.0 [139.0 - 200.0] | 0.019 |
| Charlson Comorbidity Index [0-21] | 2.0 [1.0 - 5.0] | 5.0 [2.0 - 8.0] | <0.001 |
| Diabetes mellitus | 99 (34.1) | 110 (48.0) | 0.002 |
| History of myocardial infarction | 47 (16.2) | 56 (24.5) | 0.02 |
| Left ventricular hypertrophy | 76 (26.2) | 76 (33.2) | 0.10 |
| Heart failure | 31 (10.7) | 68 (29.7) | <0.001 |
| Peripheral artery disease | 55 (19.0) | 68 (29.7) | 0.004 |
| Hypertension | 271 (93.4) | 218 (95.2) | 0.40 |
| Coronary heart disease | 88 (30.3) | 99 (43.2) | 0.002 |
| Cerebral vascular disease | 41 (14.1) | 44 (19.2) | 0.12 |
| Smoking [ever] | 79 (27.4) | 39 (19.7) | 0.051 |
| Antihypertensive medication | 258 (89.0) | 213 (93.0) | 0.11 |
| Statin | 104 (36.4) | 92 (40.2) | 0.38 |
| n (%) for categorical data, mean ± standard deviation or median [interquartile range] for continuous data, as appropriate. Abbreviations: CRP, c-reactive protein. | | | |

| **Supplemental Table 3.** Unadjusted hazards ratios of baseline parameters for cardiovascular mortality | | |
| --- | --- | --- |
| **Parameter** | **HR (95% CI)** | ***P*** |
| Age [10 years] | 1.40 (1.01-1.95) | 0.047 |
| Gender [male] | 2.12 (0.71-6.33) | 0.18 |
| Body mass index [kg/m^2^] | 1.00 (0.93-1.09) | 0.96 |
| Dialysis vintage [months] | 0.99 (1.00-1.00) | 0.84 |
| Ultrafiltration rate [ml/h] | 1.00 (1.00-1.00) | 0.21 |
| Net ultrafiltration [l] | 0.76 (0.52-1.12) | 0.16 |
| Kt/V | 0.46 (0.14-1.53) | 0.20 |
| Systolic blood pressure [mmHg] | 1.01 (0.99-1.03) | 0.59 |
| Diastolic blood pressure [mmHg] | 1.01 (0.98-1.04) | 0.46 |
| Heart rate [bpm] | 1.00 (1.00-1.00) | 0.98 |
| Blood urea nitrogen [mg/dl] | 0.98 (0.95-1.01) | 0.18 |
| Phosphate [mmol/l] | 0.87 (0.34-2.25) | 0.78 |
| Total calcium [mmol/l] | 10.5 (0.73-149.4) | 0.08 |
| Calcium *x* phosphate [mmol^2^/l^2^] | 1.02 (0.68-1.52) | 0.92 |
| Creatinine [mg/dl] | 0.87 (0.74-1.02) | 0.08 |
| High-sensitivity CRP [mg/dl] | 1.28 (1.06-1.55) | 0.011 |
| Albumin [g/dl] | 0.87 (0.28-2.72) | 0.80 |
| Parathyroid hormone [pg/ml] | 1.00 (1.00-1.00) | 0.41 |
| Leukocytes [G/l] | 1.41 (1.17-1.70) | <0.001 |
| Total cholesterol [mg/dl] | 1.00 (0.99-1.01) | 0.67 |
| High-density lipoprotein cholesterol [mg/dl]* | 1.01 (0.98-1.04) | 0.52 |
| Low-density lipoprotein cholesterol [mg/dl]* | 1.00 (0.99-1.02) | 0.53 |
| Charlson Comorbidity Index [0-21] | 1.29 (1.15-1.45) | <0.001 |
| Diabetes mellitus | 0.62 (0.26-1.49) | 0.28 |
| History of myocardial infarction | 0.13 (0.06-0.32) | <0.001 |
| Left ventricular hypertrophy | 2.10 (0.62-7.18) | 0.24 |
| Heart failure | 0.33 (0.12-0.90) | 0.030 |
| Peripheral artery disease | 0.26 (0.11-0.63) | 0.003 |
| Hypertension | 0.05 (0.00-122.32) | 0.44 |
| Coronary heart disease | 0.21 (0.09-0.53) | 0.001 |
| Cerebral vascular disease | 0.46 (0.17-1.27) | 0.14 |
| Smoking [ever] | 0.68 (0.27-1.70) | 0.41 |
| Antihypertensive medication | 0.04 (0.00-19.90) | 0.31 |
| Statin | 1.77 (0.74-4.25) | 0.20 |
| Abbreviations: CI, confidence interval; HR, hazard ratio. *Missing values n=60. | | |

| **Supplemental Table 4.** Explorative analysis of risk variables with cardiovascular mortality | | | | | | | | | | | | | |
| --- | --- | --- | --- | --- | --- | --- | --- | --- | --- | --- | --- | --- | --- |
| **Variable** | **Unit** | **Exploratory Model 5** | | **Exploratory Model 6** | | **Exploratory Model 7** | | **Exploratory Model 8** | | **Exploratory Model 9** | | **Exploratory Model 10** | |
|  |  | **HR (95% CI)** | ***P*** | **HR (95% CI)** | ***P*** | **HR (95% CI)** | ***P*** | **HR (95% CI)** | ***P*** | **HR (95% CI)** | ***P*** | **HR (95% CI)** | ***P*** |
| Age | 10 year | 1.07 (0.73-1.57) | 0.74 | 1.17 (0.79-1.74) | 0.43 | 1.13 (0.76-1.70) | 0.55 | 1.05 (0.71-1.54) | 0.82 | 1.07 (0.73-1.58) | 0.72 | 1.04 (0.70-1.55) | 0.85 |
| High-sensitivity CRP | 1 mg/dl | 1.23 (1.00-1.51) | 0.046 | - | - | - | - | - | - | - | - | - | - |
| Leukocytes | 1 G/l | - | - | 1.46 (1.17-1.81) | 0.001 | - | - | - | - | - | - | - | - |
| History of myocardial infarction | present | - | - | - | - | 6.79 (2.77-16.65) | <0.001 | - | - | - | - | - | - |
| Heart failure | present | - | - | - | - | - | - | 2.26 (0.82-6.25) | 0.12 | - | - | - | - |
| Peripheral artery diseases | present | - | - | - | - | - | - | - | - | 2.37 (0.94-5.96) | 0.07 | - | - |
| Coronary artery disease | present | - | - | - | - | - | - | - | - | - | - | 3.57 (1.42-8.98) | 0.007 |
| HRT category 1 vs 0 |  | 7.57 (1.51-37.96) | 0.01 | 8.58 (1.68-43.75) | 0.01 | 5.75 (1.19-27.87) | 0.03 | 7.48 (1.48-37.75) | 0.02 | 7.46 (1.51-36.95) | 0.014 | 6.96 (1.14-34.05) | 0.017 |
| HRT category 2 vs 0 |  | 16.98 (3.44-83.75) | 0.001 | 13.06 (2.65-64.46) | 0.002 | 16.01 (3.31-77.49) | 0.001 | 17.04 (3.41-85.17) | 0.001 | 13.56 (2.70-68.06) | 0.002 | 15.23 (3.13-74.20) | 0.001 |
| Additional exploratory models were built if a variable was predictive for cardiovascular mortality in univariate analysis. | | | | | | | | | | | | | |

| **Supplemental Table 5.** Exploratory association of HRT with cardiovascular mortality | | | | |
| --- | --- | --- | --- | --- |
| **Variable** | **Adjusted Model 3*** | | **Adjusted Model 4*** | |
|  | **HR (95% CI)** | ***P*** | **HR (95% CI)** | ***P*** |
| HRT category 1 vs 0 | 7.76 (1.53-39.22) | 0.013 | 6.86 (1.37-34.36) | 0.019 |
| HRT category 2 vs 0 | 17.49 (3.49-87.51) | <0.001 | 11.41 (2.19-59.58) | 0.004 |
| *Note: Due to the presence of 5 and 6 variables in relation the 20 cardiovascular mortality events in adjusted Model 3 and 4, respectively, this model could potentially be overfitted.  Adjusted Model 3: Age, albumin, high-sensitivity CRP, calcium *x* phosphate, HRT.  Adjusted Model 4: Age, albumin, high-sensitivity CRP, calcium *x* phosphate, Charlson Comorbidity Index, HRT.  Abbreviations: CI, confidence interval; HR, hazard ratio; HRT, heart rate turbulence. | | | | |

| **Supplemental Table 6.** Unadjusted and adjusted hazards ratios of ln transformed frequency-domain heart rate variability measures for cardiovascular mortality | | | | | | | |
| --- | --- | --- | --- | --- | --- | --- | --- |
| **Cardiovascular mortality risk** | | | | | | | |
| **Parameter** | **Unit** | **Univariate** | | **Adjusted Model 1** | | **Adjusted Model 2** | |
|  |  | **HR (95% CI)** | ***P*** | **HR (95% CI)** | ***P*** | **HR (95% CI)** | ***P*** |
| Total Power | ln(ms^2^) | 0.67 (0.35-1.30) | 0.24 | 0.72 (0.36-1.42) | 0.34 | 0.95 (0.49-1.85) | 0.89 |
| ULF | ln(ms^2^) | 0.68 (0.36-1.28) | 0.23 | 0.74 (0.39-1.40) | 0.35 | 0.95 (0.51-1.78) | 0.87 |
| VLF | ln(ms^2^) | 0.70 (0.41-1.20) | 0.19 | 0.75 (0.43-1.29) | 0.30 | 0.92 (0.52-1.62) | 0.78 |
| LF | ln(ms^2^) | 0.72 (0.46-1.11) | 0.14 | 0.71 (0.45-1.12) | 0.14 | 0.81 (0.51-1.31) | 0.40 |
| HF | ln(ms^2^) | 0.94 (0.63-1.41) | 0.75 | 0.82 (0.53-1.26) | 0.36 | 0.91 (0.58-1.42) | 0.67 |
| Model 1: age, albumin; model 2: age, Charlson Comorbidity Index. Abbreviations: CI, confidence interval; ULF, ultra-low frequency; VLF, very low frequency; LF, low frequency; HF, high frequency. | | | | | | | |

| **Supplemental Table 7.** Association of severe autonomic failure (SAF) with cardiovascular mortality in univariate and multivariable analysis | | | | | | | |
| --- | --- | --- | --- | --- | --- | --- | --- |
| **Variable** |  | **Univariate** | | **Adjusted Model 1** | | **Adjusted Model 2** | |
|  |  | **HR (95% CI)** | ***P*** | **HR (95% CI)** | ***P*** | **HR (95% CI)** | ***P*** |
| Severe autonomic failure 1 vs 0 |  | 5.95 (2.47-14.36) | <0.001 | 5.11 (2.09-12.49) | <0.001 | 3.36 (1.25-9.03) | 0.016 |
| Adjusted model 1 includes: Age, albumin and severe autonomic failure. Adjusted model 2 includes: Age, Charlson Comorbidity Index and severe autonomic failure. Severe autonomic failure is assumed if HRT category 2 and deceleration capacity ≤ 4.5 ms are present (n=36). Abbreviations: CI, confidence interval; HR, hazard ratio | | | | | | | |

| **Supplemental Table 8.** Ln transformed frequency-domain measures of heart rate variability at baseline in patients overall and grouped by survival state | | | | |
| --- | --- | --- | --- | --- |
| **Parameter** | **Unit** | **Overall** | **Cardiovascular mortality** | |
|  |  |  | **Survivor (n=270)** | **Non-survivor (n=20)** |
| Total Power | ln(ms^2^) | 8.95±0.69 | 8.96±0.69 | 8.78±0.57 |
| ULF | ln(ms^2^) | 8.76±0.72 | 8.77±0.72 | 8.58±0.65 |
| VLF | ln(ms^2^) | 6.48±0.82 | 6.50±0.83 | 6.23±0.65 |
| LF | ln(ms^2^) | 5.56±0.98 | 5.58±1.00 | 5.21±0.69 |
| HF | ln(ms^2^) | 4.74±0.98 | 4.75±1.09 | 4.63±0.89 |
| Values are expressed as mean ± standard deviation. ULF, ultra-low frequency; VLF, very low frequency; LF, low frequency; HF, high frequency. | | | | |

**Supplemental Figure 1.** Cumulative cardiovascular mortality curves stratified by heart rate turbulence (HRT) including HRT category non-calculable.

The number of patients of the individual groups involved in the analysis at 1, 2 and 3 years are shown under the graph. 3-year cardiovascular mortality rates were 0.9%, 1.9%, 10.2%, and 22.0% for those with HRT category non-calculable (n.c.), 0, 1, and 2, respectively. There was no significant difference in the survival probability of patients in HRT category 0 and HRT category non-calculable (χ^2^=0.25, log-rank p=0.62).
